# A new multi-epitope DNA vaccine against Helicobacter Pylori infection in a BALB/c mouse model

**DOI:** 10.1016/j.heliyon.2024.e39433

**Published:** 2024-10-17

**Authors:** Zahra Azami, Mahnaz Farahmand, Mahsa Kavousi

**Affiliations:** Department of Biology, East-Tehran Branch, Islamic Azad University, Tehran, Iran

**Keywords:** Bioinformatics analysis, *H. Pylori*, In-silico, Novel vaccine

## Abstract

**Background:**

*Helicobacter Pylori* (*H. Pylori*) is a pathogen that may invade the human stomach. This bacterial strain is now causing widespread concern and considerable health issues worldwide. In contrast to antibiotic treatment, which may lead to drug resistance, vaccination therapy is emerging as a possible immunotherapy option for *H. Pylori*. DNA vaccines are a potential option to traditional vaccines among vaccine research methods. Furthermore, the multiepitope DNA vaccination may induce a broader immune response to suppress *H. Pylori* infection.

**Methods:**

Four target antigenic proteins (outer membrane beta-barrel, outer membrane beta, HofA, and hcp beta-lactamase-like protein) were used to identify epitopes. The best B and T cell epitopes were selected to induce humoral and cellular immune responses and were connected using the HEYGAEALERAG and GGGS linkers. The peptide's physicochemical characteristics, secondary and tertiary structures, antigenicity, and allergenicity were evaluated utilizing several bioinformatics tools. The multiepitope peptide was successfully inserted into the pcDNA3.1 expression vector. The immunological responses of both the vaccinated and control groups were evaluated by measuring cytokines and antibodies.

**Results:**

Based on the data, the multiepitope peptide consists of 278 amino acid residues and has an average molecular weight (MW) of 28643.61 Da. The peptide residues were mainly situated within the preferred and permitted areas of the Ramachandran plot, accounting for 92.86 % of the total. The VaxiJen server has calculated that the multiepitope peptide has an antigenicity score of **1.0067**. BALB/c mice vaccinated with the DNA vaccine produced significantly higher levels of specific IgG antibodies (*p < 0.05*). The vaccinated mice exhibited a TH1-type cellular immune response characterized by the generation of IFN-γ and a longer length of life compared to the control animals (*p < 0.05*). In addition, the vaccination group exhibited a substantial increase in the expression level of IFN-γ and IL-1β genes compared to the control group (*p < 0.05*).

**Conclusions:**

The results demonstrated that the multiepitope DNA vaccine elicited significant humoral and cellular responses, and increased survival time in BALB/c mice, indicating that selecting potential epitopes may be a viable technique for developing multiepitope-based vaccines. This can help to introduce effective vaccines.

## 1 Introduction

*H. Pylori*, a prevalent gram-negative bacterium, has a very high infection rate, affecting around 50 % of the global population. It is categorized as a Class I carcinogen (IARC) by the World Health Organization (WHO) and the International Agency for Research on Cancer [1]. *H. Pylori* infection has been associated with several gastrointestinal disorders, such as chronic gastritis, peptic ulcers, gastric malignancies, as well as non-gastric illnesses such as iron deficiency anemia, idiopathic thrombocytopenic purpura, and nonalcoholic-fatty-liver [2]. If the infection continues, the sickness will progress, possibly resulting in malignant conditions. Managing *H. Pylori* mainly involves using proton pump inhibitors, antibiotics, and bismuth treatments. However, the development of biofilms by *H. Pylori* to reduce its vulnerability to certain drugs makes eradicating more difficult. Furthermore, *H. Pylori* infection can reoccur after undergoing eradication treatment, resulting in financial and physical discomfort for patients [3]. Therefore, designing a secure, reliable, and efficient vaccination that targets several epitopes and can induce an immune response against *H. Pylori* infection is crucial.

Vaccine development requires the first step of screening for antigen targets. Previous studies have shown that specific antigens, such as urease, neutrophil-activating protein (NAP), and superoxide dismutase (SOD), are very suitable candidates for eliciting a defense reaction versus *H. Pylori* [4,5]. Generally, it is necessary to acknowledge that confirmed antigens have a restricted impact on eliminating *H. Pylori* in the stomach [6]. Forecasting novel candidate antigens via reverse vaccination is essential to conduct high-throughput screening. Bioinformatics-driven approaches have shown success in reverse vaccination strategies for a range of illnesses, such as Serogroup B meningococcus [7], Klebsiella pneumonia [8], and Mycobacterium abscesses [9]. The reverse vaccination technique offers a more favorable option than traditional methods, as it may expedite the discovery of novel antigens and reduce the occurrence of antigenic failures.

There has been a particular emphasis on creating recombinant multiepitope vaccines in the field of vaccine design and development. A multiepitope vaccine consists of B and T epitopes derived from different antigens ordered in a precise sequence, together with appropriate linkers that may inhibit the creation of junctional epitopes and enhance the process of antigen presentation [10]. The creation of several epitopes has been shown to provide benefits by activating a wider protective the immune system's reaction and effectively blocking various pathogenic pathways for controlling *H. Pylori*. This approach is distinct from the administration of single, unaltered antigen vaccinations. Recent experiments have shown that several multiepitope vaccinations versus *H. Pylori* may elicit substantial quantities of specific antibodies versus different epitope-producing antigens [11,12].

To analyze the immunogenicity of a target protein, it is essential to identify the epitopes that immune cells may present to elicit an immune system reaction. T-cell epitopes and B-cell epitopes are two distinct forms of epitopes. T cell epitopes are presented by the MHC molecule and recognized by T cells, whilst B cell epitopes are recognized by immunoglobulin [13]. Based on the structural properties, conformational epitopes were found to be made up of non-continuous portions in the sequence, yet they are tightly packed together in space. Linear epitopes refer to the specific peptides within protein sequences with functional properties. This work examined linear epitopes, namely MHC-binding peptides and B-cell linear epitopes, to develop multiepitope vaccines [14].

In this study, we selected four antigenic proteins (Outer membrane beta-barrel, outer membrane beta, HofA, and hcp beta-lactamase-like protein) for the computational design of a multi-epitope-based vaccination model. A comprehensive computational methodology was used to develop a vaccine against pathogenic *H. Pylori* by identifying shared antigenic B-cell and T-cell epitopes. The HofA protein, an outer membrane protein, is a promising candidate for an *H. Pylori* infection vaccine. The *H. Pylori* hcp β protein is categorized as either β-lactamases or cysteine-rich proteins and has pathogenic characteristics [15].

For this investigation, we selected four *H. Pylori* antigenic proteins: Outer membrane beta-barrel, outer membrane beta, HofA, and hcp beta-lactamase-like protein. By analyzing these antigenic proteins, researchers found shared epitopes on B-cells and T-cells. Subsequently, a vaccination formulation was developed. In addition, we have found many possible qualities of the vaccine, such as secondary structure, antigenicity, protein solubility prediction, and physiochemical properties. Furthermore, using the reverse vaccinology approach, the immunogenicity of the vaccine was assessed in BALB/c mice. The indirect ELISA test was used to quantify the levels of total IgG in order to evaluate the humoral immune response. The cytokines IL1-b and IFN-γ expression levels were assessed using real-time PCR and ELISA methods. The present work aims to create a potent multiepitope peptide vaccine component targeting *H. Pylori*.

## 2 Methods

### 2.1 In silico analysis

#### 2.1.1 Screening of potential vaccine proteins using bioinformatics

Four proteins—outer membrane beta-barrel, outer membrane beta, HofA, and hcp beta-lactamase-like protein—were selected for the development of a computer-aided multiepitope peptide vaccine versus *H. Pylori* based on their functions in *H. Pylori* infection. The criteria for epitope recognition for T and B cells were high solubility, presence in the outer membrane, lack of homology with host proteins, possession of antigenic characteristics, and interactions with other proteins. The UniPort service (https://www.uniprot.org/) was used to verify the existence of several proteins in the *H. Pylori* proteome. The amino acid sequences of these proteins were retrieved from NCBI (http://www.ncbi.nlm.nih.gov/protein) in FASTA format for further analysis. The proteins were assessed for this objective employing the host protein Homo sapiens/mice and the BLASTp online tool(https://blast.ncbi.nlm.nih.gov/Blast.cgi?PROGRAM=blastp&PAGE_TYPE=BlastSearch&LINK_LOC=blasthome) on the NCBI website. The CELLO program (http://cello.life.nctu.edu.tw/) was used to ascertain the exact localization of proteins inside the *H. Pylori* bacteria. One may determine if a protein is located in the cytoplasm or periplasm by examining the location of molecules outside the cell using a Localization Reliability score of 1.5 on either the outer or inner membrane. Proteins necessary for a bacterium's functioning are referred to as essential proteins. These proteins are considered potential targets for vaccines and play a substantial role in the activity of the bacteria. The DEG database (http://www.essentialgene.org) contains proteins experimentally identified as crucial proteins in the pathogen. Therefore, the DEG server examined the antigen proteins [16].

#### 2.1.2 Investigating the antigenic properties of selected proteins in H. Pylori

The VaxiJen tool (http://www.ddg-pharmfac.net/vaxijen/VaxiJen/VaxiJen.html) was used to forecast the antigenicity of the possible immunogenic targets. A cut-off value of 0.5 was utilized. The Protein-Sol server (http://proteinsol.manchester.ac.uk) was used to assess the solubility of the multiepitope vaccine. The projected solubility refers to the value of solubility that has been adjusted or scaled based on the QuerySol value [17].

#### 2.1.3 T cell and B cell epitope prediction

T-cells are crucial in initiating cytotoxic and helper T-cell-mediated immune system reactions. The IEDB MHC binding prediction tool (https://tools.iedb.org/mhci/) was used to predict MHC-I and MHC-II epitopes. The IEDB linear epitope prediction algorithm (v2.0) used the default settings to forecast B-cell epitopes. This program utilizes a sophisticated algorithm derived from the three-dimensional protein structures of antigen-antibody complexes. The investigation was conducted on the Bepipred 2.0 web server (https://services.healthtech.dtu.dk/services/BepiPred-2.0/) provided by the IEDB [18].

#### 2.1.4 Prediction of tertiary structure

Homology modeling is constructing a detailed model of a specific protein utilizing its arrangement of amino acids and the known structures of similar proteins, referred to as templates. The SWISS-MODEL server (http://swiss-model.expasy.org) is a fully automated platform for homology modelling protein structures. QMEAN, also known as Quantitative Model Energy Analysis, is a scoring function that quantifies the essential geometric properties of protein structures. The quality of all 3D protein models generated by the SWISS-MODEL service [19] was assessed using QMEAN scores.

#### 2.1.5 B-cell epitope prediction and screening

Determining B-cell epitopes is crucial in creating vaccines, immunodiagnostic testing, and producing antibodies. The Ellipro service (http://tools.immuneepitope.org) was used to forecast linear antibody epitopes by leveraging the 3D structure of a protein target. Furthermore, the DiscoTope 2.0 Server (https://services.healthtech.dtu.dk/services/DiscoTope-2.0/) predicted B-cell structural epitopes [20]. Epitopes with a maximum length of residues were ultimately chosen.

#### 2.1.6 Construction of model vaccine

Final vaccine development often selects common B-cell and T-cell epitopes based on their strong immunogenicity and lack of allergenicity. In order to create a chimeric vaccine, the chosen epitopes (HTL, CTL, and B epitopes) were joined together using amino acid linkers (HEYGAEALERAG and GGGS). Adjuvants (C274) were included in the structures to enhance the immune response. The linker "EAAAK" was added at both ends (N and C) to increase immunogenicity further. The genetic sequence of this adjuvant is 5′-tcgtcgaacgttcgagatgat-3'.

#### 2.1.7 Antigenicity, allergenicity, and physiochemical properties of the designed vaccine

The antigenic capacity of the vaccination model was evaluated utilizing the VaxiJen v2.0 server. Furthermore, the AlgPred server (http://crdd.osdd.net/raghava/algpred/) was used to evaluate allergenicity. The Protein-Sol website (https://protein-sol.manchester.ac.uk/) was utilized to assess the solubility of the vaccine design. In order to assess the hydrophilicity, heat resistance, and stability of the protein vaccine, it is necessary to examine the GRAVY, aliphatic index, and protein instability index (<45), respectively. The Expasy server (https://www.expasy.org/) was employed for this objective [19].

#### 2.1.8 In silico cloning and codon optimization

The vaccine construct underwent codon optimization utilizing the Java Codon Adaptation Tool (JCAT), an open web server. This was done to enhance the generation of the vaccine sequence at a high level in the E. coli K12 strain. The pcDNA3.1(+) vector was used for in silico cloning of the vaccination sequence. The RF-Cloning.org program creates the final vaccine construct in the pcDNA3.1(+) vector. This construct includes prokaryotic ribosome binding sites, separate transcription terminators, multiple enzyme restriction sites and the gene sequence necessary for vaccine production [21].

### 2.2 In vitro and in vivo analysis

#### 2.2.1 Bacterial strains and growth environments

The *H. Pylori* strain (ATCC: 43504) was provided by the Iranian Biological Resource Center (IBRC). The bacteria were propagated for 72 h at 37 °C on a Columbia agar medium supplemented with 10 % horse serum and dent supplement (Oxoid, UK) under controlled conditions of reduced oxygen (5 % O2), increased carbon dioxide (10 % CO2), and nitrogen (85 % N2) at 95 % humidity. This was achieved using an anaerobic jar and a Campygen GasPak system (Merck, USA). In order to prepare bacteria for animal injection, a single colony was placed in Trypticase Soya broth (Condalab, Spain). The bacteria were cultured for 7 h under microaerophilic conditions and adjusted to around 107 Colony Forming Units (CFU) [22]. The E. coli TOP10F and pcDNA3.1 (+) plasmids utilized for the research were procured from Novagen Technology (Beijing, China) and were cultivated in LB broth medium for one night at a temperature of 37 °C.

#### 2.2.2 Plasmid construction, purification, and expression

The final sequence of the multiepitope vaccine has been delivered to a Beijing firm (Shenzhen, China) for manufacturing and cloning into the eukaryotic expression vector pcDNA3.1, as mentioned before. The C274 adjuvant sequence was produced and included before the target gene inside the recombinant plasmid. The Shenzhen business supplied the synthesized recombinant plasmid. To generate adequate plasmids for future research, the recombinant vector was introduced into Escherichia coli strain Top10 F using a process including treatment with CaCl2 (0.1 M) and heat shock at 42 °C for 90 s. PCR was used to confirm the existence of vector transformation. The colonies containing the modified vector were grown overnight in LB broth supplemented with 100 μg/mL of ampicillin antibiotic, stirring at 180 rpm at a temperature of 37 °C. The GeneJET Plasmid Miniprep Kit from Thermo Fisher Scientific in Freiburg, Germany, was used to isolate plasmids. Subsequently, colony-PCR, sequencing, and enzymatic digestion using *Bam*HI and *Eco*RV restriction enzymes were performed on the purified plasmids to demonstrate successful transformation. Following the manufacturer's guidelines, the MaxiPrep Plasmid Purification Kit (Qiagen) was used for large-scale plasmid extraction. The concentration of multiepitope plasmid was determined using a spectrophotometer at wavelengths of 260 and 280 nm [23].

#### 2.2.3 Animals

The AmitisGen Tech Dev group (Tehran, Iran) provided female BALB/c mice that were 6–8 weeks old and had a body weight of 20 ± 5 g. These animals were maintained under standard lighting, feeding, and watering conditions. The study was approved by the Ethics Committee of the Islamic Azad University-East Tehran Branch in Tehran, Iran.

#### 2.2.4 Immunization and challenge

40 female BALB/c mice were randomly assigned to four groups (n = 10): empty plasmid (pcDNA3.1), PBS, recombinant multiepitope vaccination, and recombinant multiepitope vaccine with C 274 adjuvant. Table 1 shows that on days 0, 7, and 15, 100 μg of plasmid was administered into the quadriceps muscle of mice using 100 μl of saline phosphate buffer. To test the efficiency of multiepitope DNA vaccines versus *H. Pylori*, all groups were challenged with 1 × 10^9^ CFU *H. Pylori* four weeks following their previous vaccination (on day 45). Survival was observed for seven days to evaluate interventions. Healthy and sick mice were investigated at different time intervals (days 7, 14, and 21 post-gavage). The animals were examined for changes in fur, body weight, and survival rate after being treated with several vaccinations vs control mice.

**Table 1:** Grouping and sampling of treated models.

Groups	Injection time (days)	Sampling
pcDNA3.1	0, 7, 15	Whole blood, Quadriceps muscle, Spleen
PBS	0, 7, 15	Whole blood, Quadriceps muscle, Spleen
Multiepitope vaccine + C274	0, 7, 15	Whole blood, Quadriceps muscle, Spleen
Multiepitope vaccine	0, 7, 15	Whole blood, Quadriceps muscle, Spleen

#### 2.2.5 Evaluation for colonization of H. Pylori in gastric tissue after the challenge

The stomach tissue was first isolated to determine the level of *H. Pylori* colonization in the stomachs of the mice that were challenged. Subsequently, the tissue was bisected along its central axis. The PBS-washed portion was employed to extract the whole DNA via the supernatant. Subsequently, the PCR test was conducted. The remaining portion of the stomach tissue underwent staining examinations. To determine the presence of *H. Pylori* colonization in the stomach tissue of the mice and assess the rate of positive *H. Pylori* infection and protection, PCR, urease test, and Gram staining techniques were used.

#### 2.2.6 Expression of DNA vaccines in mice

The TRIzol reagent (Invitrogen, Carlsbad, CA, USA) from Thermo Fisher Scientific was used to separate total RNA from the injected muscle tissues. Gene-specific primers were generated using the Oligo7 bioinformatics program. The primers were validated using the BLAST online tool on the GenBank website, maintained by the National Center for Biotechnology Information (NCBI) in the United States. The Sequence Match program from the Ribosomal DatH database, namely the pyloriase Project of the RDP II in the USA, was also used for validation. The mice's thigh tissue (injection site) was assessed for the relative mRNA expression level of DNA vaccines using RT-PCR. Following the YTA Kit (Yekta Tajhiz, Iran) protocol, cDNA synthesis was performed. After DNase treatment, cDNA synthesis was performed by combining 1 μL of oligo (dT), 1 μL of dNTP Mix (10 mm per dNTP), about 1 μg of RNA, and nuclease-free water in a 0.2 mL PCR tube. The total volume was adjusted to 13 μL. The mixture was then incubated at 65 °C for 5 min and then chilled on ice for at least 1 min. The tube underwent a treatment at a temperature of 50 °C for 60 min. The treatment included adding 1 μL of 0.1 m dithiothreitol (DTT), 1 μL of SuperScript III Reverse Transcriptase, 4 μL of 5 × First-Strand Buffer, and 1 μL of RNaseOUT Recombinant RNase Inhibitor. Following deactivation at a temperature of 70 °C for 15 min, the tube underwent treatment at 37 °C for 20 min using 1 μL of RNase H. The GAPDH gene was an internal reference in the RT-PCR procedure (Table 2). The PCR result was evaluated with agarose gel electrophoresis.

**Table 2:** The primer sequences used in q-RT-PCR.

Gene name	Sequence (3’→5′)	TM (°C)	Size (bp)
Multi epitope vaccine	F: 5′-CGACCAAAGAAGAACCGAAA-3′	58	211
	R: 5′-CTATCATACGCCAGGCTGCT-3′		
*GAPDH*	F: 5′- TGTGTCCGTCGTGGATCTGA-3′	60	78
	R: 5′- CCTGCTTCACCACCTTCTTGA-3′		
*IL1-b*	F: 5′- AATCTCGCAGCAGCACATCAAC -3′	60	195
	R: 5′- CCAGCAGGTTATCATCATCATCC -3′		
*IFN-γ*	F: 5′- AGCGGCTGACTGAACTCAGATTGTAG-3′	60	199
	F: 5′- GTCACAGTTTTCAGCTGTATAGGG-3′		
*16SrRNA*	F: 5′- GAGACACGGTCCAGACTCCT-3′	60	200
	F: 5′- CTTGCACCCTCCGTATTACC-3′		

#### 2.2.7 In-vitro analysis of DNA vaccines in E. coli BL21 (DE3) using Western blot analysis

Using the Western blotting technique and the antigenic sandwich methodology, the Rojan Azma Group (Iran) investigated antigen purification to assess antigen synthesis. Both control group samples and recombinant DNA vaccine samples were collected, separated using electrophoresis on 12 % SDS PAGE gels, and then applied onto a nitrocellulose membrane (GE Amersham Biosciences, USA). The proteins were identified by Western blotting at a temperature of 4 °C for one night. Monoclonal antibodies targeting the r-multi epitope were employed at a concentration 1/1000, obtained from Santa Cruz Biotechnology, USA. The antibodies were coated with nitrocellulose layers and then tagged with HRP. The first Abs were labeled and identified utilizing anti-Abs, HRP (Santa Cruz Biotechnology), and ECL reagents.

#### 2.2.8 Immunological analysis

The Enzyme-Linked Immunosorbent Assay (ELISA) kits were used to assess Total IgG, IL-1β, and IFN-γ cytokine levels. The whole blood sample was collected and centrifuged with a force of 10,000 times the acceleration due to gravity for 10 min. The resulting serum was then stored at a temperature of −20 °C until it was required. The detection of H.pylori-specific IgG antibodies in BALB/c mouse blood samples was performed using commercially available enzyme-linked immunosorbent assay (ELISA) kits, following the instructions provided by the vendor. According to the manufacturer's instructions, the cytokine production was assessed using IL-1β and IFN-γ Mouse ELISA kits (Karmania ParsGen, Iran). An ELISA reader was used to measure the optical density (OD) of several samples at a wavelength of 450 nm. The cut-off value of the ELISA was calculated by calculating a 2.1-fold increase above the average OD value of negative serum samples from normal, unimmunized mice. The trials were conducted three times [24].

#### 2.2.9 Splenocyte activation and secretion of cytokines

Splenocytes were harvested from immunized mice precisely seven days after the last administration of the vaccine. After adjusting the splenocyte density to 1 × 106 cells/mL, 200 μL of the fluid culture was added to a 96-well plate. Subsequently, the splenocytes were either stimulated with a DNA vaccine at 20 μg/mL concentration or left without stimulation. Following a 72-h incubation period, the levels of IFN-γ and IL-1β in the liquid above the cells were measured using mouse IFN-γ and IL-1β ELISA kits.

#### 2.2.10 Investigating the expression of cytokines with qRT-PCR

The TRIzol reagent (Invitrogen, USA) was used to extract total RNA from 100 mg of each spleen tissue sample, following the manufacturer's instructions. The RNA samples were quantified by measuring their absorbance at 260–280 nm wavelengths. Subsequently, cDNA samples were synthesized using a cDNA synthesis kit (Biotechrabbit, Germany) with specific primers or combined with an oligo (dT) primer, following the instructions provided by the manufacturer. Gene Runner software, version 3.05, and the BLAST of GenBank data (Table 2) were used to generate precise oligonucleotide primers. The expression levels of cytokine genes, such as IL1-b and IFN-γ, were evaluated using GAPDH (glyceraldehyde 3-phosphate dehydrogenase) as a reference gene. The cDNA samples, measuring 0.5 μl each, were diluted in a ratio of 1:10 and added to a final volume of 15 μl. This final volume comprised 10 μl of SYBR Green PCR Master Mix from ThermoFisher in Germany and 0.5 μl of each primer. The process began with an initial denaturation step at a temperature of 95 °C for 10 min. This was followed by 40 cycles of denaturation at the same temperature (95 °C) for 20 s. The next step included annealing the primers at the temperature in Table 2 for 40 s. Finally, an extension step was performed at 72 °C for 30 s. The reactions were conducted twice, and the q-RT-PCR was assessed by measuring fluorescence during the annealing phase of each cycle. The standard curve approach was used to determine the relative amounts of gene expression. The study focused on analyzing the cycle of the threshold (Ct) values of the target and reference genes using the comparative Ct (2^-△△ Ct^) approach [25].

### 2.3 Statistical analysis

GraphPad Prism 5.0 was used for data analysis and statistical testing. The Tukey post hoc technique was used to do multiple comparisons among the groups. The unpaired *t*-test was used to compare the survival rates of vaccinated mice with those of the control group. A unidirectional analysis of variance (ANOVA) was used to compare means, followed by a Tukey-Kramer post hoc test at a 95 % confidence level. Statistical significance was determined at a threshold of p < 0.05.

## 3 Results

### 3.1 In silico

#### 3.1.1 Target protein identification

Four *H. Pylori* proteins were assessed in this study for potential applications in vaccine development. After verifying the existence of four proteins in *H. Pylori* through the Uniport database, the accession numbers of these proteins were chosen from the NCBI database. Using the CELLO software, their locations in the extracellular, outer, periplasmic, internal, and cytoplasmic regions were determined and recorded in Table 3. The following proteins were obtained in FASTA format from the NCBI database.

• Outer membrane beta-barrel protein (accession number: WP 020995799.1)
• Outer membrane beta (accession number: WP 212869334.1)
• HofA (accession number: WP 223894332.1)
• Hcp beta-lactamase-like protein (accession number: BAW57543.1)

**Table 3:** The initial screening identified proteins with high solubility, flexibility, and antigenic score as suitable for *H. Pylori* vaccine candidates.

Protein name	ACCESSION NUMBER	Location (CELLO analysis)		Protein. Sol	BlastP		Antigenicity
Outer membrane beta-barrel protein	WP_020995799.1	Extracellular	1.638 ∗	0.55	**Humo sapiens (taxid:9606)**	**No significant similarity found**	**0.6946**
					**mouse (taxid:****10088****)**	**No significant similarity found**	
Outer membrane beta	WP_212869334.1	Extracellular	2.580 ∗	0.62	**Humo sapiens (taxid:9606)**	**No significant similarity found**	**0.6030**
					**mouse (taxid:****10088****)**	**No significant similarity found**	
HofA	WP_223894332.1	Outer Membrane	4.217 ∗	0.51	**Humo sapiens (taxid:9606)**	**No significant similarity found**	0.4642
					**mouse (taxid:****10088****)**	**No significant similarity found**	
Hcp beta-lactamase-like protein	BAW57543.1″ title = "ncbi-p:BAW57543.1">BAW57543.1	Extracellular	1.501 ∗	0.52	**Humo sapiens (taxid:9606)**	**No significant similarity found**	0.6098
					**mouse (taxid:****10088****)**	**No significant similarity found**	
∗ Statistically significant							

The BLASTp results confirmed that most of the selected proteins were highly specific for *H. Pylori*, which decreases the chances of the vaccine causing a response in host cells.

#### 3.1.2 Identification of MHC-I and MHC-II epitopes

The proteins described were evaluated for their antigenic properties and subjected to epitope analysis using the IEDB server. Based on the findings from the SOLpro tool, the proteins described have been verified to have appropriate solubility for epitope analysis, as shown in Table 3. The capacity of *H. Pylori* to link MHC-II/MHC-I proteins' epitope sites to distinct HLA was expected, given that the bacteria is an extracellular bacterium that may also operate intracellularly under stressful situations. The relationship between the epitope sites and the related alleles is better when this server's adjusted rank value is lower. The adjusted rank range of various HLA alleles for MHC-I and MHC-II was determined based on this data, as shown in Table 4. In addition, the immunogenicity of the MHC peptide complex (pMHC) was predicted using the Class I Immunogenicity tool. Class I Immunogenicity prediction utilizes the amino acid characteristics and their specific location within the peptide. Table 4 displayed the 5 alleles that exhibited the highest affinity for the specified proteins and the immunogenicity of class I proteins.

**Table 4:** The IEDB server predicts B cell, MHC-I and MHC-II binding epitopes, with adjusted rank estimating survival function. Closer numbers indicate greater protection.

Protein	Binding Prediction	selected epitopes	Start	End	Peptide	Adjusted rank
Outer membrane beta-barrel protein	MHC.I	HLA-A∗01:01	6	15	NTDSFTLGAY	0.01
		HLA-A∗26:01	23	31	HVINVGYSY	0.01
		HLA-A∗68:02	56	64	ATFAGSHKV	0.04
		HLA-A∗02:03	14	25	AYVGFGLGY	0.11
		HLA-B∗15:01	34	43	IDMFLGDMNY	0.44
	MHC.II	HLA-DQA1∗01:01/DQB1∗05:01	21	35	GYGITGITDQKAAID	0.01
		HLA-DRB1∗15:01	9	23	SFTLGAYVGFGLGYG	0.65
		HLA-DRB1∗07:01	1	15	MVNFLNTDSFTLGAY	0.70
		HLA-DQA1∗01:02/DQB1∗06:02	48	62	IPINVGIAATFAGSH	0.81
		HLA-DQA1∗04:01/DQB1∗04:02	39	53	GDMNYNGFNIPINVG	1.60
	B cell	–	5	9	LNTDS	–
		–	24	32	ITGITDQKA	–
		–	41	47	MNYNGFN	–
		–	73	87	LSAGYSSKTKNDKSE	–
	Selected allele	NTDSFTLGAY (MHCI, MHCII)				–
		GYGITGITDQKAAID (MHCII)				
		LSAGYSSKTKNDKSE (MHCII and B cell)				
Outer membrane beta	MHC.I	HLA-A∗23:01	41	49	SYFQMPVEF	0.01
		HLA-A∗23:0	24	32	IYFNYMINF	0.01
		HLA-B∗15:01	33	41	ALQKPSHVF	0.01
		HLA-A∗03:01	56	64	ALYNFYESK	0.02
		HLA-B∗35:01	2	10	NAQGLSSAF	0.04
	MHC.II	HLA-DQA1∗01:02/DQB1∗06:02	37	51	VHKNIQTAVAQAQAT	0.01
		HLA-DRB3∗02:02	19	33	YGYFSYNHANLSFVG	0.01
		HLA-DRB1∗11:01	31	45	KDAFMNVHKNIQTAV	0.04
		HLA-DRB3∗02:02	48	62	EFGFRSNFSKRSGIE	0.22
		HLA-DRB1∗01:01	1	15	TNQFYKERGVDGSVD	0.56
	B cell	–	45	86	AAPTKEEPKKTASGEPTPSTPPTKKDETSDSGSTPSSSGSSV	–
		–	215	224	TGSSTNSTTT	–
		–	256	273	AQAQATYKPSVMNTNNYG	–
		–	343	348	SKDGYN	–
	Selected allele	SYFQMPVEF (MHCI)				–
		SFKKLGFVSLATSSV (MHCII)				
		AAPTKEEPKKTASGEPTPSTPPTKKDETSDSGSTPSSSGSSV (B cell)				
HofA	MHC.I	HLA-B∗07:02	32	40	SPRANSQSL	0.01
		HLA-B∗57:01	42	50	ITHDTKSYW	0.01
		HLA-B∗58:01	19	27	FVFQRVHFR	0.02
		HLA-B∗07:02	13	21	DIFGIKLGR	0.02
		HLA.A∗24:02	51	60	KSGWRIQTTF	0.05
	MHC.II	HLA-DPA1∗02:01/DPB1∗01:01	17	31	SLAYDSTKFLIDEAD	0.01
		HLA-DRB3∗02:02	31	45	SFSRIAFNQSVINSK	0.12
		HLA-DQA1∗01:01/DQB1∗05:01	49	63	NGSPIDPFYDTKDDT	0.21
		HLA-DRB1∗04:01	51	65	KSGWRIQTTFYAWFP	0.28
		HLA-DRB1∗13:02	41	55	GVTFKYNIKKHIYLM	0.43
	B cell	–	47	56	GIYPTGSYVT	–
		–	127	137	NWTREQKAQNA	–
		–	319	350	FGNGSAQLGWNGSPIDPFYDTKDDTPYEDAYS	–
		–	427	435	GYNPDFAQT	–
	Selected allele	ITHDTKSYW (MHCI)				–
		SLAYDSTKFLIDEAD(MHCII)				
		FGNGSAQLGWNGSPIDPFYDTKDDTPYEDAYS (B cell)				
hcp beta-lactamase-like protein	MHC.I	HLA-A∗03:01	16	25	KSYEKQDFSK	0.03
		HLA-B∗40:01	3	12	AEPNPEELVL	0.05
		HLA-A∗26:01	58	66	DSKKAVALF	0.1
		HLA-B∗44:02	21	29	QDFSKARKY	0.11
		HLA-A∗24:02	28	36	KYFEKACDL	0.28
	MHC.II	HLA-DRB1∗12:01	7	21	PEELVLLGIKSYEKQ	0.02
		HLA-DRB1∗03:01	52	66	GDGVKQDSKKAVALF	0.23
		HLA-DPA1∗03:01/DPB1∗04:02	15	29	IKSYEKQDFSKARKY	0.96
		HLA-DPA1∗02:01/DPB1∗14:01	25	39	KARKYFEKACDLNNG	2.10
		HLA-DRB3∗01:01	46	60	GGLYYNGDGVKQDSK	2.30
	B cell	–	19	25	EKQDFSK	–
		–	50	61	YNGDGVKQDSKK	–
	Selected allele	KSYEKQDFSK (MHCI)				–
		PEELVLLGIKSYEKQ (MHCII)				
		EKQDFSK, YNGDGVKQDSKK (B cell)				

#### 3.1.3 Prediction of B cell epitopes

**Fig. 1:**
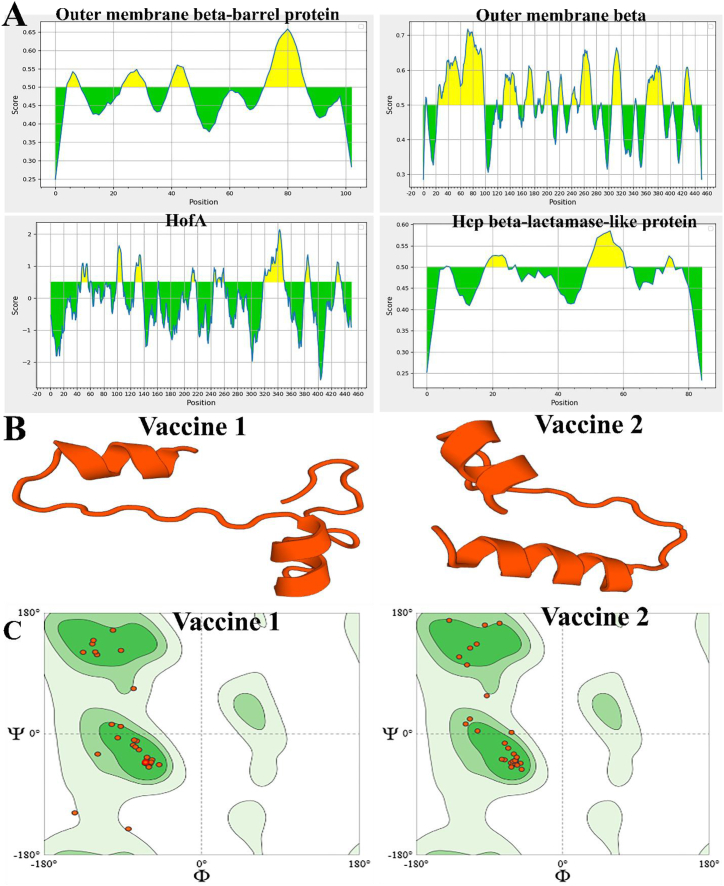
**A)** Identification of B cell epitopes for each protein. **B)** 3D structure of vaccine constructs 1 and vaccine construct 2. **C)** Ramachandran plot of vaccine1 structure showed its more stability than vaccine 2 structure.

The IEDB online program was used to evaluate the linear B cell epitopes. The findings indicated that the proteins listed had at least four epitopes. The density of epitopes in B cells was determined using the Bepipred Linear Epitope and Bepipred Linear Epitope Prediction 2.0 software, respectively, with a threshold of 0.500 (Fig. 1A).

#### 3.1.4 Antigenicity, allergenicity, and physiochemical properties of the developed vaccine

**Fig. 2:**
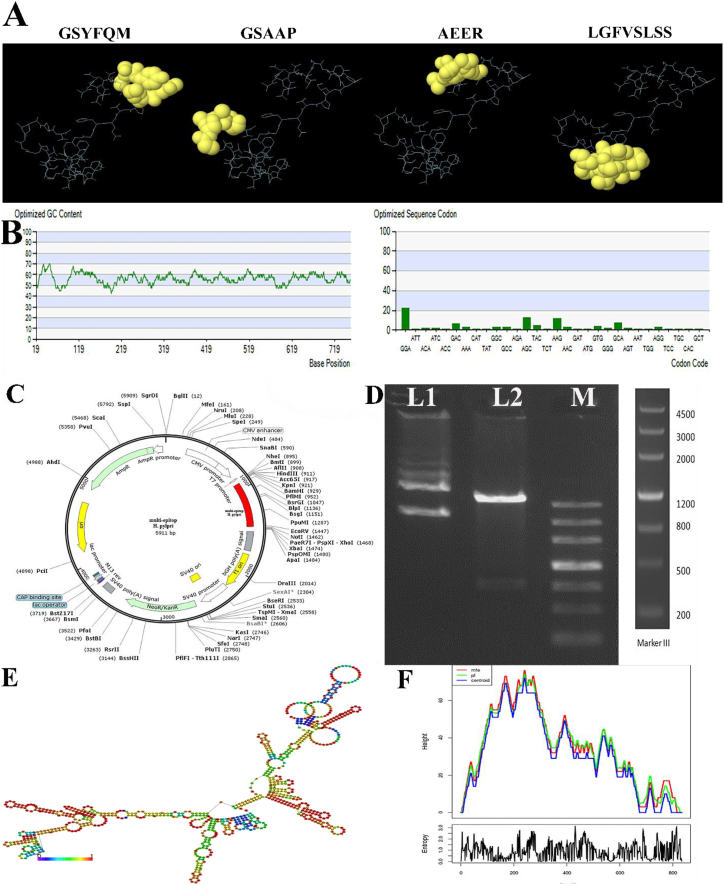
**A)** Predicted Discontinuous Epitope for vaccine 1. **B)** The analysis of codon optimization in the vaccine structure reveals its optimization specifically for mouse muscle. **C)** Vaccine cloning using computational methods. The RED region inside the vector represents the multi-epitope vaccination insert. **D)** Electrophoretic pattern of digestion products and purified plasmids on a 1 % agarose gel. The pure pcDNA3.1(+) undigested (L1), purified pcDNA3.1(+) (L2), and DNA ladder (M) were subjected to digestion using *Bam*HI and *Eco*RV restriction enzymes. **E)** mRNA structures predicted from the vaccine structure. **F)** The mRNA entropy of the vaccine structure indicates the mRNA stability.

The antigenic propensity of a vaccine construct is an essential metric for creating vaccine constructs that normally generate both humoral and cell-mediated immune responses against *H. Pylori.* According to the results (Table 5), 2 vaccine structures with antigenic properties of **1.0067** and **0.7810** were identified, the first structure was selected as a vaccine candidate with suitable solubility, negative GRAVY index, non-allergenic, and stability due to the suitable molecular weight for predicting the secondary structure. The 3D structure of the 2-vaccine structure was obtained and shown in Fig. 1B. The vaccine 1, with a Ramachandran Favored proportion of 92.86 % and no Bad Bonds (0/313), was selected because of its high stability. Vaccine 2 had a well-organized composition, with 96.97 % of its Ramachandran angles favorably positioned and no instances of unfavorable chemical bonds (0 out of 261). However, vaccination 2 was removed from the trial because it contained 4 instances of poor angles (4/349) at A96 PHE, A110 GLU, and A83 GLU residues, and its Rotamer Outliers percentage was 4.17 % at A85 LEU residue (Fig. 1C). Predicted Discontinuous Epitope for vaccine 1 was shown in Fig. 2A.

**Table 5:**
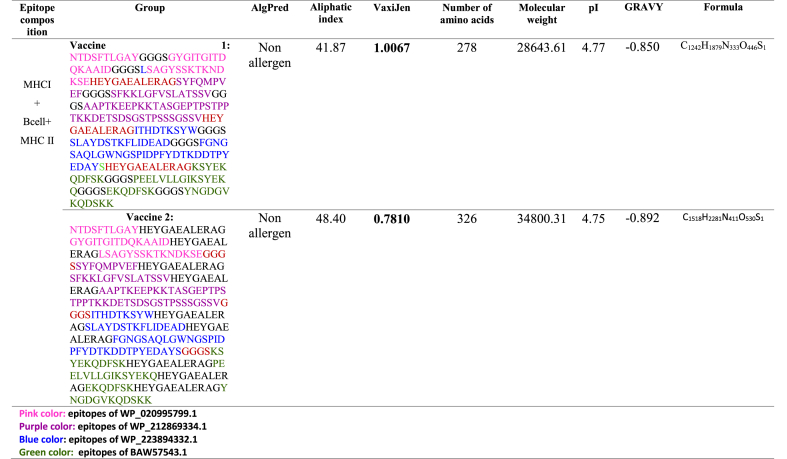
The main sequence of a multiepitope vaccine construct is evaluated considering factors such as antigenicity, allergenicity, solubility, and physicochemical properties.

#### 3.1.5 In silico cloning and optimization of codon

In order to understand the production of our designed multiepitope vaccine in E. coli hosts, we performed in silico cloning. The Java Codon Adaptation (JCat) tool enhanced our methodology for optimizing gene expression in the E. coli K12 strain. The optimal vaccine design has a CAI value of 0.92, with a range of 0.8–1.0. The optimum GC content range is 63.67 %, ranging from 30 to 70 %. These values suggest a high likelihood of protein expression, as shown in Fig. 2B. During the subsequent stage, we include two restricted endonucleases (*Bam*HI and *Eco*RV) at both ends of the vaccine construct to facilitate the cloning process. The absence of cleavage of the double vaccination sequence in the pcDNA3.1 expression vector was verified using *Bam*HI and *Eco*RV enzymes for both the vaccine and C274 adjuvant, as determined by the GenScript server. The DNA sequences of *Bam*HI (GGATCC) and *Eco*RV enzymes were inserted at the 5′ and 3′ termini of the fused vaccine construct, respectively. The clone had a size of 5911 kilobytes, as seen in Fig. 2C. The vaccine design map is displayed in Fig. 2C. Electrophoretic pattern of digestion products and purified plasmids on a 1 % agarose gel was shown in Fig. 2D. The improved vaccine construct was inserted into the pcDNA3.1 cloning plasmid using the SnapGene program. The RNA fold server was used to predict the secondary structure of the mRNA. The minimum free energy, which was −280.30 kcal/mol, indicates the thermodynamic stability of the mRNA structure. Additionally, the first 12 nucleotides of the mRNA secondary structure did not have pseudoknots or elongated stable hairpins, making it easier to start translation from the mRNA framework (Fig. 2E). A mountain plot was used to depict the MFE structure, the thermodynamic ensemble of RNA structures, and the centroid structure. Furthermore, the positional entropy is shown for each location (Fig. 2F).

### 3.2 In vitro and in vivo

#### 3.2.1 Cloning, expression, and confirmation of the pcDNA3.1 (+)/CPG

The chemically produced gene was effectively inserted into pcDNA3.1 by cloning. Colony PCR was conducted on many transformed colonies with chimeric gene-specific primers. The positive randomly selected plasmids were verified by enzyme digestion, 1 % agarose gel electrophoresis, and sequencing. The existence of a segment in the recombinant vector was confirmed using restriction analysis (Fig. 2D). The digestion products were successfully separated by electrophoresis at 834 base pairs, demonstrating the effective production of the recombinant plasmid. The proper cloning and synthesis of the pcDNA3.1(+)/CPG recombinant plasmid is confirmed by the presence of an 834 bp band after digestion with *Bam*HI (GGATCC) and *Eco*RV (GATATC) enzymes. Additionally, the nanodrop device was used to ascertain the concentration of the extracted plasmids, which was found to be 694.4 ng/μl for every 5 μL of the plasmid.

#### 3.2.2 Expression of recombinant plasmids in mRNA and protein levels

**Fig. 3:**
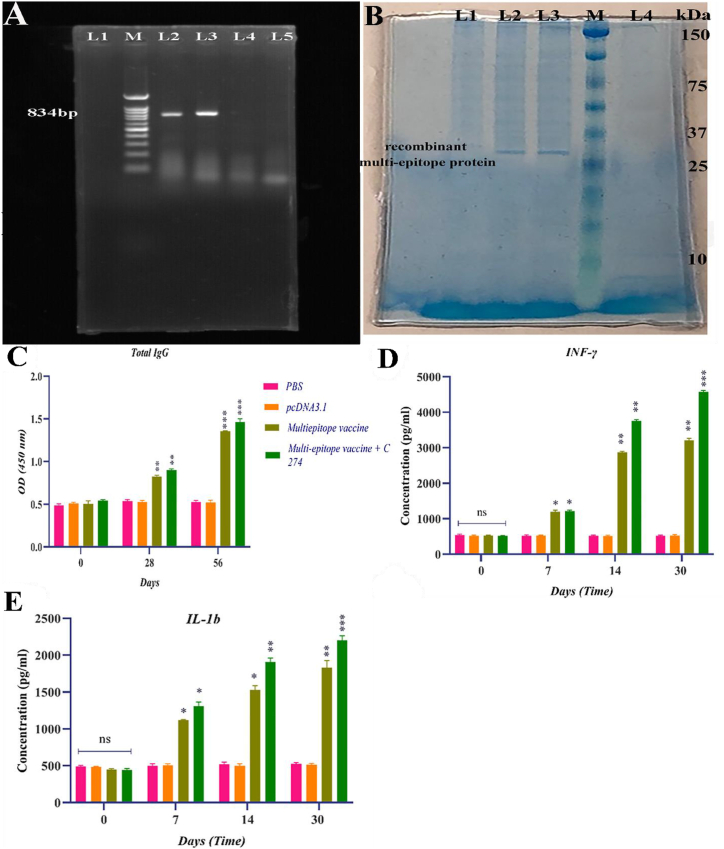
**A)** Analysis of mRNA expression levels of recombinant multiepitope DNA vaccine. L1: negative control; M:100 bp DNA ladder; L2: multiepitope vaccine, L3: Multiepitope vaccine + C274; L4: pcDNA3.1 and L5: PBS. **B)** Production of recombinant multiepitope vaccine at the protein level. L1: PBS; L2: multiepitope vaccine, L3: Multiepitope vaccine + C274; L4: pcDNA3.1 and M: protein marker. **C)** The total IgG antibodies in the sera of BALB/c mice were measured after vaccination with different formulation. **D)** Assessment of IFN-γ and **E)** IL-1β cytokines was conducted in the splenocytes of immunized mice. The values are shown as the mean ± SD (∗*p < 0.05*, ∗∗p < 0.01 and ∗∗∗p < 0.001).

The mRNA expression levels of the multiepitope DNA vaccines were evaluated using reverse transcriptase-PCR (RT-PCR). A prominent band in the agarose gel confirms the effective transference of the targeted plasmid into the host cell. Fig. 3A suggests that DNA vaccines' capacity to infiltrate host cells is limited, as shown by other groups with less potent bands. Rojan Azma's Western blot analysis revealed the production of the target proteins at the protein level. The recombinant multiepitope protein, with a molecular weight of 28643.61 (28 kDa), was successfully produced, as shown by the presence of the antigen at the protein level (Fig. 3B).

#### 3.2.3 Humoral immune responses

The mice were then immunized intramuscularly with the recombinant vaccine. The sera derived from the hearts of mice were examined using ELISA for this specific objective. Mice inoculated with the multiepitope vaccine, with or without CPG/C274, exhibited significantly elevated IgG antibody titers compared to the control groups (PBS and empty pcDNA3.1 vector) 30 days after the last injection (P ≤ 0.01). Furthermore, the findings indicated that the overall IgG concentration in the group that only received the unbound plasmid was not substantially different from the control group (PBS). The average total IgG levels in the groups that received the vaccine at different periods validate the increase in the immune response during the immunization period. In order to evaluate the antibody-mediated immune response under various vaccination schedules, BALB/c mice were administered with several DNA vaccine formulations, each containing 100 μg, by subcutaneous delivery. The animals were administered PBS and pcDNA3.1 as the negative and positive control groups. Following the administration of the third injection, serum samples were collected from the mice that had been inoculated.

We conducted a study to explore the capacity of the multi-epitope to enhance the immunological function of antibodies in animals. This was achieved by evaluating the performance of r-multi epitope-specific antibodies using the enzyme-linked immunosorbent assay (ELISA). Fig. 3C demonstrates that the serum from the negative and positive control groups included a considerably higher amount of IgG antibodies than those present after vaccination with the recombinant multi-epitope. This difference was statistically significant with a p-value of less than 0.01 (P < 0.01). The PBS control group's blood samples did not contain antibodies specific to the antigen (P > 0.05).

#### 3.2.4 Cytokines production

In order to evaluate the production of IFN-γ and IL-1β cytokines, splenocytes were collected from three BALAB/c mice in each group seven weeks after the last vaccination. The levels of these cytokines were determined using ELISA. Fig. 3D and E shows that spleen cells from mice inoculated with the multiepitope vaccine generated significantly higher levels of IFN-γ and IL-1β compared to animals in the PBS and empty pcDNA3.1 vector groups (P < 0.05). Furthermore, the groups that received adjuvant treatment had a more robust cellular immune response than the others, as shown by elevated levels of functional cytokines (IFN-γ, IL-1β, P < 0.01).

#### 3.2.5 Splenocyte Induction and cytokine analysis

On the seventh day after the third immunization, splenocytes were obtained from six mice in each group and subjected to DNA vaccine activation in culture. The enzyme-linked immunosorbent assay (ELISA) was used to detect the production of interleukin-1β (IL-1β) and interferon-gamma (IFN-γ) in the culture supernatants. Fig. 3E demonstrates a disparity in IL-1β production between the DNA vaccine group and the control groups. On the other hand, Fig. 3D reveals that the splenocytes from the mice infected with the DNA vaccine produced significantly higher levels of IFN-γ compared to the other group (P < 0.01).

#### 3.2.6 IFN-γ, IL-1β gene expression analysis by RT-qPCR

**Fig. 4:**
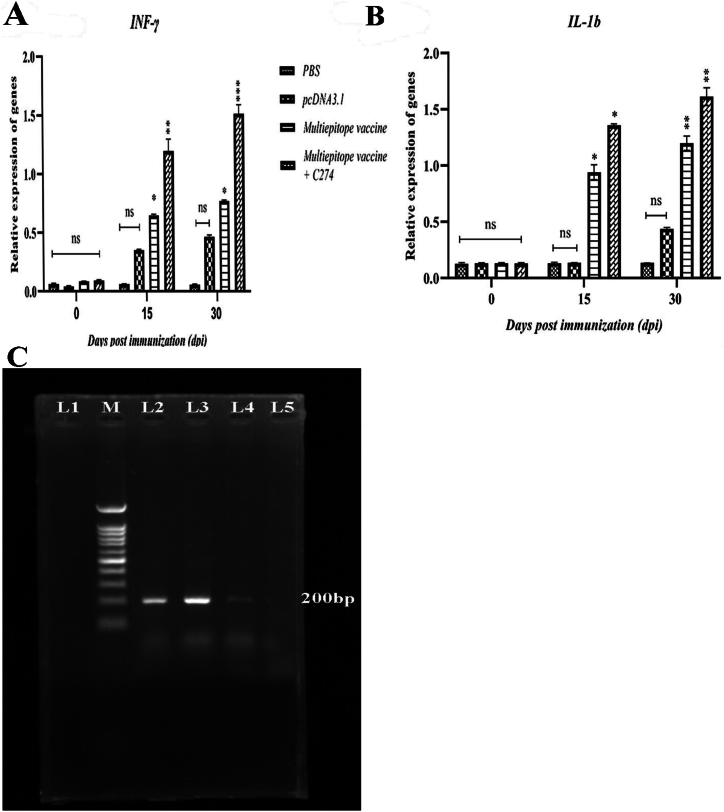
**(A)** IFN-γ and **(B)** IL-1β gene expression in the spleens of BALB/c mice given a 100 μg Multiepitope vaccine + C274. qRT-PCR was used to identify the messenger RNA (mRNA) for the IFN-γ and IL-1β genes. Data are presented as mean ± SD. The relative quantification was performed by the comparative Ct method (ΔΔCt), using the GAPDH as the reference gene. ns; not significant, ∗*P < 0.05*; ∗∗P < 0.01; ∗∗∗P < 0.001. **C)** The results of the PCR test on the stomach tissue of the challenged mice showed that the 200bp band was clearly present in the PBS and pcDNA3.1 groups. L1: Negative control, L2: PBS, L3: pcDNA3.1, L4: Multiepitope vaccine, L5: Multiepitope vaccine + C274 and M: 100bp DNA ladder.

The gene expression of interleukin-1b (IL-1β) and Interferon-gamma (IFN-γ) was assessed in the splenocytes of the vaccinated group both before and after the challenge. This was done to investigate the potential association between the cellular immune response induced by vaccination and the production of cytokines. The findings indicated that there was no statistically significant difference (P > 0.05) in the expression of IL-1β and IFN-γ genes between the group that received pcDNA3.1(+) throughout the vaccination period and the PBS control group. Analysis of cytokine gene expression levels in the spleens of infused BALB/c mice showed significant variation in the expression levels of IFN-γ and IL-1β genes between the Multiepitope vaccine + C274 group and the PBS group (P < 0.01). The expression of the IL-1β and IFN-γ genes was significantly increased in the BALB/c mice that received multiepitope vaccination and Multiepitope vaccine + C274 on days 15 and 30 (P < 0.01) (Fig. 4A and B).

#### 3.2.7 Results for colonization of H. Pylori

The PCR test findings on the stomach tissue of the challenged mice revealed the presence of a distinct 200bp band in the PBS and pcDNA3.1 groups, confirming bacterial colonization in these groups. However, a band of extremely low intensity was seen in the group that received the Multiepitope vaccination. In addition, it is worth noting that no discernible band was present in the Multiepitope vaccine + C274 group. This observation shows the vaccine's efficacy in preventing colonization, as seen in Fig. 4C. In addition, the gram staining test conducted on the control groups revealed the existence of gram-negative bacilli in the stomach tissue. Conversely, these bacteria were absent in the vaccinated groups. We used the rapid urease test (RUT), a conventional diagnostic method for detecting *H. Pylori*. A gastric antrum was used to get a mucosa biopsy, which was then immersed in a solution containing urea and a phenol red indicator. The pH of the environment increased in the PBS and pcDNA3.1 groups due to the action of urease produced by *H. Pylori*, which converts urea into ammonia. This caused a change in the color of the sample from yellow (indicating a negative result) to red (indicating a positive result). The color of the environment did not change in the Multiepitope and Multiepitope vaccine + C274 groups, indicating the high efficacy of these vaccinations in preventing *H. Pylori* colonization.

## 4 Discussion

*H. Pylori* is a parasitic bacterium that resides in the stomach and duodenum of humans and may lead to infection if not eradicated [26]. The removal of *H. Pylori* significantly reduces stomach inflammation and promotes the healing of ulcers. Nevertheless, since the antibiotic resistance of *H. Pylori* continues to increase, eliminating the bacteria becomes more complex, and there is a risk of the infection returning even after eradication medication. Therefore, there is an urgent need for the development of a potential vaccine against *H. Pylori*. Scientists are using immunoinformatic techniques to build a vaccine for preventing *H. Pylori* infection. This approach is being used since conventional vaccine development methods have certain limits and drawbacks, such as being time-consuming and inefficient [2,3]. No adequate and efficient vaccine candidate is available to treat *H. Pylori* infection [2]. The current study aims to develop a peptide vaccine against *H. Pylori* using a multiepitope-based approach. An epitope-based vaccination has the advantage of explicitly stimulating immune responses against epitopes. It also enhances the binding affinity with target receptor molecules, preventing any adverse effects from non-epitope selection [27]. We used a contemporary immunoinformatic method to select four antigenic proteins (outer membrane beta-barrel protein, outer membrane beta, HofA, and Hcp beta-lactamase-like protein) for the creation of a novel multiepitope vaccine targeting *H. Pylori*. This study identified T-cell epitopes that originated from B-cell epitopes, which were demonstrated to contribute to developing both humoral and cellular immune responses. Subsequently, various web-based techniques were used to accurately forecast B cell linear, MHC-I, and MHC-II epitopes. Linkers were used to establish connections between epitopes and incorporate them into other chimaera components [28].

Research on naturally occurring proteins with many domains has provided inspiration for the development of artificial protein fusion using linker peptides. Linkers in multiepitope vaccines provide notable benefits, including reducing the probability of junctional antigen generation and improving antigen processing and presentation. However, the role of the linker in structural flexibility and stiffness is crucial [29]. The research used HEYGAEALERAG and GGGS protein linkers. GGGS made a versatile contribution. The proteasomal system (HEYGAEALERAG) and lysosomal system (HEYGAEALERAG) were chosen as cleavage sites.

In recent years, researchers have explored many methods to enhance the immunogenicity of DNA vaccines. These methods include using adjuvants, altering codon bias, cytokines and chemokines, utilizing CpG, electroporation, liposomes, microparticles, and heterologous prime/boost vaccination [30]. Prior studies have shown that the effectiveness of DNA vaccines mainly relies on the existence of unmethylated CpG motifs in the plasmid backbone, which function as an inherent adjuvant. Plasmid DNA that contains CpG motifs or synthesized CpG oligonucleotides (CpG ODNs) stimulates the proliferation, maturation, and release of various cytokines, chemokines, and immunoglobulins in B cells, NK cells, and monocytes/macrophages [[31], [32], [33]]. Considering the functional importance of the CpG adjuvant (5′-tCGtCGaaCGttCGagatgat-3′), we used EAAAK to enhance rigidity, hence reducing the likelihood of other protein segments interfering with the interaction between the adjuvant and its receptor. Per the ExPASy ProtParam online tool, the created peptide consists of 322 amino acid residues and has an average molecular weight of 28 kDa (kDa). Peptides having molecular weights over 10 kDa are classified as immunogenic [34]. Essentially, a high aliphatic index signifies that the peptide is more resistant to changes in temperature and remains stable across a broad range of temperatures. A negative value for the GRAVY parameter indicates that the peptide interacts more strongly with the water molecules in its environment [35].

The primary biological function of peptides is closely related to their spatial arrangements. The ultimate objective of protein structure prediction is to predict the tertiary structures of peptides [36] accurately. In this work, the SWISS-MODEL online server was used to predict the three-dimensional structure of the multiepitope vaccine. Next, we used the Ramachandran plot to validate the generated 3D model using the SWISS-MODEL service. Based on the estimated results, the baseline model included more than 90 % of all residues corresponding to recommended and authorized regions. Epitope analysis is an essential process for determining the antigenicity of peptide antigens, which in turn provides insights into the activities and structures of the antigen.

Moreover, epitope analysis sheds new insights into the pathophysiology of diseases and immunological mechanisms, enabling scientists to create vaccines based on epitopes [37]. Accurately identifying potential epitopes depends on several protein structural features rather than a single one. However, evaluating a single index must provide more precise information about the desired sequence. Epitope determination frequently involves evaluating many characteristics, such as flexibility, hydrophilicity, antigenicity, and surface accessibility. Consequently, a high-index peptide may easily bind to antibodies and be considered a suitable epitope. The research used the IEDB online program to detect linear epitopes of B lymphocytes. Epitopes are more likely to be identified when the peptide score is higher.

The evaluations of allergenicity and antigenicity demonstrated that the multiepitope vaccine is devoid of allergenic properties and capable of inducing an immune response. Consequently, we used this multiepitope vaccine to evaluate its efficacy and ability to stimulate an immune response against acute *H. Pylori* infection. In this investigation, BALB/c mice were administered a multiepitope DNA vaccine by subcutaneous injection, which resulted in protective immunity against acute *H. Pylori* infection. Our findings indicate that a multiepitope DNA vaccine stimulated both humoral and cellular responses. The objective is to create a multiepitope vaccine that may generate a wide-ranging antibody response, effectively inhibiting several pathogenic routes of various pathogens. Humoral immunity controls *H. Pylori* infection by producing targeted IgG antibodies. This specific reaction results in the suppression of *H. Pylori* colonization and harmful effects. This experiment revealed elevated IgG antibody levels in the vaccinated mice serum. The addition of the C274 adjuvant may significantly enhance the IgG response.

Multiple research investigations indicate that antibodies may play a role in vaccine-induced immunity, although they are unnecessary for immunological protection. Alternatively, several studies suggest that a humoral immune response is necessary for eradicating *H. Pylori* [27].

The significance of CD4^+^ T cells in protecting *H. Pylori* has been acknowledged. Therefore, a successful *H. Pylori* vaccination should generate efficient CD4^+^ T cell responses [38]. CD4^+^ T cells, or Th cells, differentiate into T helper 1 (Th1) or T helper 2 (Th2) cells based on their cytokine production patterns. Th1 cells mostly create IFN-γ and IL-2, while Th2 cells mainly generate IL-4, IL-5, IL-10, and IL-13 [39]. While Th1-type responses are involved in the pathophysiology of *H. Pylori* infection, the specific involvement of Th1 or Th2 in vaccine-induced protection is still debatable [38]. It has been suggested that both Th1 and Th2 cells have a role in defending against *H. Pylori* infection [39]. IL-1β strongly inhibits the formation of stomach acid and has a crucial role in initiating and intensifying the inflammatory response to *H. Pylori* infection [40]. IL-1β is a vital cytokine in the gastrointestinal system that has diverse biological effects on many tissues. It has a role in inflammatory, metabolic, physiologic, hematopoietic, and immunological processes. IL-1β is generated at elevated levels in myeloid cell lineages in response to tissue injury and microbial invasion [41].

In addition, numerous types of cells, such as B cells, T cells, NK cells, dendritic cells, fibroblasts, and epithelial cells, also secrete this protein in response to different stimuli and under inflammatory conditions, although to a lesser extent [42,43]. Increased IL-1β-driven inflammation of the stomach mucosa may speed up the removal of germs and limit gastric acid production. These are essential responses to *H. Pylori* infection [40]. The ELISA test findings demonstrated that the levels of IFN-γ and IL-1β in the multiepitope and multiepitope vaccination with C274 adjuvant groups were markedly elevated compared to the control group. In addition, the real-time PCR data demonstrated an upregulation of IFN-γ and IL-1β gene expression in the groups who received the multiepitope vaccination. Adding the CpG-C274 adjuvant to the multiepitope vaccination group may substantially enhance the level of IFN-γ. The findings indicated that mice immunized with the multiepitope DNA vaccine had a Th1 immune response, as shown by significant levels of IFN-γ and IgG. Multiple investigations have shown that *H. Pylori* infections result in mice's mortality within 15–30 days following exposure [[40], [41], [42], [43]]. Researchers have made substantial progress in developing DNA vaccines to combat *H. Pylori* infection in recent decades, primarily using computational and laboratory approaches. For instance, Junfei Ma et al. used in silico analysis to choose Omp6, UreB, and PLA1 from *H. Pylori* as appropriate candidates for designing a multiepitope vaccine [4]. Immunity was enhanced by using a unique construction method to develop multiepitope vaccines. These vaccines were formulated using PLA1, Omp6, and UreB epitope analyses [4,44,45]. Based on our research, their study presents robust principles for constructing epitopes in developing multiepitope vaccines, which may be advantageous in reducing *H. Pylori* infection in the future. This research suggests that significant progress is still needed in developing an efficacious immunization against *H. Pylori*. Further research in this field may aid in unravelling the vaccination conundrum for this particular pathogen. Concurrently, DNA immunizations are showing potential.

## 5 Conclusion

*H. Pylori* is a very prevalent bacterium worldwide. Immunoinformatic approaches were used to develop a novel multiepitope vaccine against *H. Pylori*, including outer membrane beta-barrel protein, outer membrane beta, HofA, and Hcp beta-lactamase-like protein. Various online tools were used to verify the vaccine's stability, expression capacity, and antigenicity. Additionally, in silico cloning was utilized to confirm the stability and expression. Analysis in the field of bioinformatics has shown that specific peptides possess a multitude of potential epitopes for both B and T-cells. Furthermore, the linear B-cell epitopes exhibited favorable characteristics regarding surface accessibility, flexibility, hydrophilicity, and antigenicity. This peptide, consisting of several epitopes, exhibited both immunogenicity and non-allergenicity. At vivo studies have shown that this DNA vaccine, which targets several epitopes, has successfully stimulated immune solid responses at both the humoral and cellular levels. Additionally, it has been seen that the vaccine has extended the lifetime of BALB/c mice. These findings suggest that selecting potential epitopes might be viable for developing a multiepitope-based vaccination.

## CRediT authorship contribution statement

**Zahra Azami:** Writing – review & editing, Writing – original draft, Validation, Software, Methodology, Formal analysis. **Mahnaz Farahmand:** Writing – review & editing, Writing – original draft, Supervision, Methodology. **Mahsa Kavousi:** Writing – review & editing, Writing – original draft, Methodology, Investigation, Funding acquisition.

## Ethical approval

Not required.

## Funding

This research did not receive any specific grant from funding agencies in the public, commercial, or not for profit sectors.

## Declaration of competing interest

The authors declare that they have no known competing financial interests or personal relationships that could have appeared to influence the work reported in this paper.
