# Supplementary material for: A new multi-epitope DNA vaccine against Helicobacter Pylori infection in a BALB/c mouse model
Source: Heliyon. 2024 Oct 17;10(21):e39433. doi: 10.1016/j.heliyon.2024.e39433 (PMC11546231; doi:10.1016/j.heliyon.2024.e39433)
Supplement: Multimedia component 1 [file mmc1.docx]

**Supplementary Information**

| 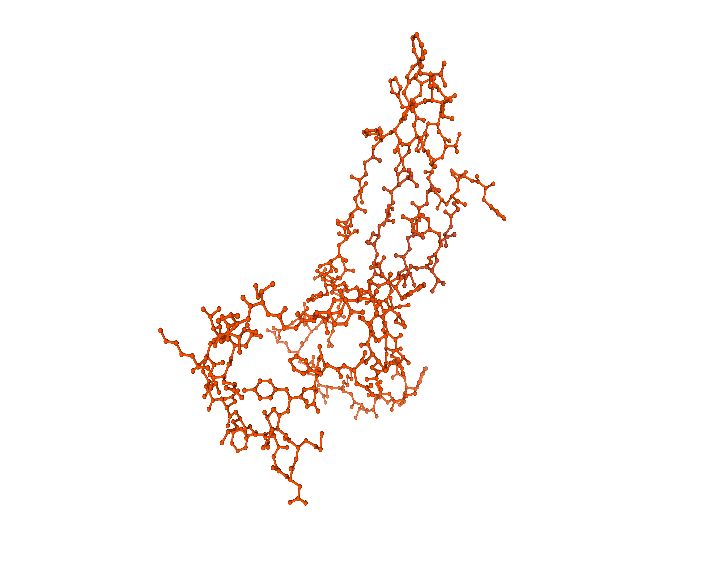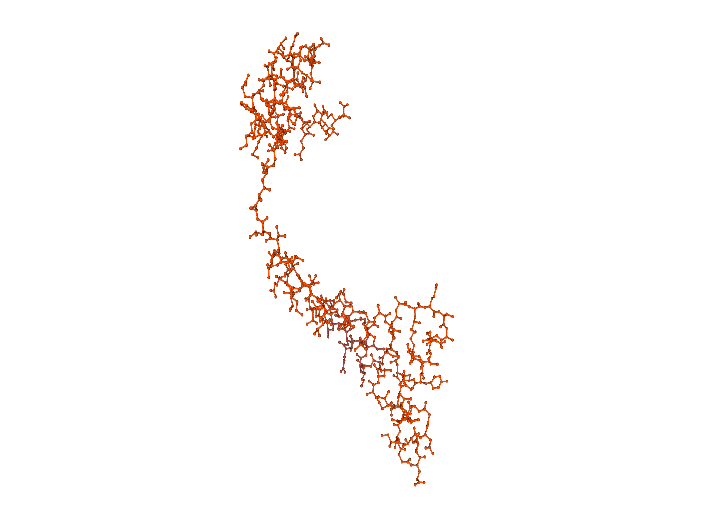  **B**  **A**    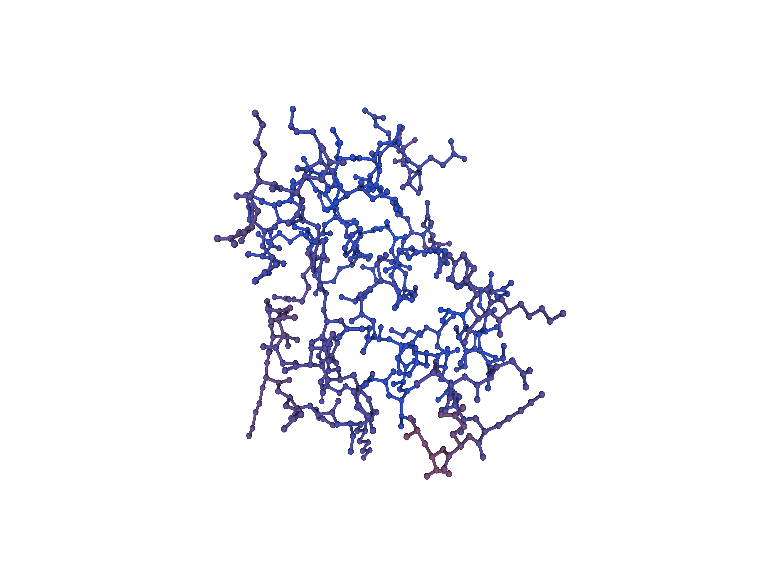  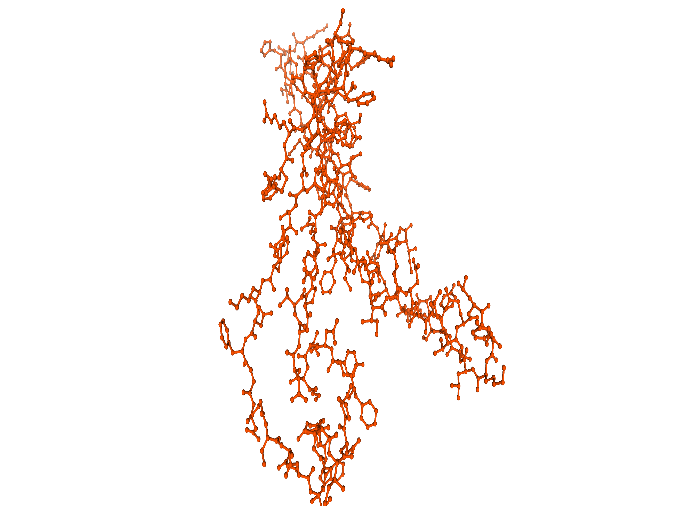  **D**  **C**    **Figure S1.** The 3D structures of outer membrane beta-barrel protein (A), outer membrane beta (B), HofA (C), and Hcp beta-lactamase-like protein (D) predicted by SWISS-MODEL tool. |
| --- |

| 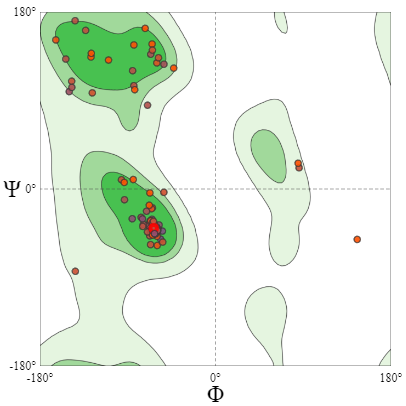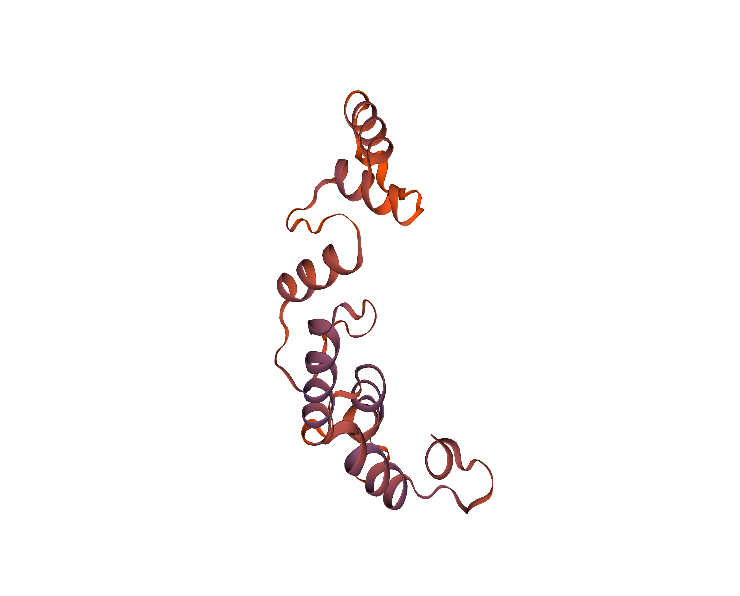  **B**  **A**    **C**  GITGITDQK HEYGAEALERAG LSAGYSSKTKNDKSE GGGS SYFQMPVEF GGGS SFKKLGFVSLATSSV GGGS FNQSVINSKKGI GGGS KFLIDEADPKA GGGS QKAQNA GGGS VNYKQKTN GGGS KSYWK GGGS YRASLIG GGGS FYDTKDDT GGGS YSPRA GGGS YNPDFAQTIQN GGGS KQDFSK GGGS KQDFSKARKY GGGS QDSKKAVALF GGGS GLYYNGDGV GGGS KQDFSKARKYFEKAC GGGS EKQDFS GGGS KQDFSKARKYFEKAC GGGS VKQDSKKAVALFEKA HEYGAEALERAG QDFSKARKYFEKACD  **Figure S2.** Structure modeling and validation of vaccine. (A, B) The 3D model and Ramachandran plots of a multiepitope vaccine was obtained by Swiss-model, (C) vaccine sequence. |
| --- |
